# Effect of luteal-phase support on endometrial microRNA expression following controlled ovarian stimulation

**DOI:** 10.1186/1477-7827-10-72

**Published:** 2012-09-06

**Authors:** Yulian Zhao, Howard Zacur, Chris Cheadle, Ning Ning, Jinshui Fan, Nikos F Vlahos

**Affiliations:** 1 Department of Gynecology and Obstetrics, Johns Hopkins University School of Medicine, Lutherville, MD, USA; 2 Department of Medicine, Johns Hopkins University School of Medicine, Baltimore, MD, USA; 3 Department of Obstetrics and Gynecology, The First Affiliated Hospital, Harbin Medical University, Harbin, China; 4 2nd Department of Obstetrics and Gynecology, Aretaieion Hospital, University of Athens School of Medicine, Athens, Greece

**Keywords:** MicroRNA, Ovarian stimulation, Luteal phase support, Microarray

## Abstract

**Background:**

Studies suggested that microRNAs influence cellular activities in the uterus including cell differentiation and embryo implantation. In assisted reproduction cycles, luteal phase support, given to improve endometrial characteristics and to facilitate the implantation process, has been a standard practice. The effect of different types of luteal phase support using steroid hormones in relation to endometrial miRNA profiles during the peri-implantation period has not seen described. This study was designed to evaluate the expression of miRNAs during the luteal phase following controlled ovarian stimulation for IVF and the influence of different luteal phase support protocols on miRNA profiles.

**Methods:**

The study was approved by the Johns Hopkins Hospital Institutional Review Board. Endometrial biopsies were obtained on the day of oocyte retrieval from 9 oocyte donors (group I). An additional endometrial biopsy was obtained 3–5 days later (Group II) after the donors were randomized into three groups. Group IIa had no luteal-phase support, group IIb had luteal support with micronized progesterone (P), and Group IIc had luteal support with progesterone plus 17-beta-estradiol (P + E). Total RNA was isolated and microarray analysis was performed using an Illumina miRNA expression panel.

**Results:**

A total of 526 miRNAs were identified. Out of those, 216 miRNAs were differentially regulated (p < 0.05) between the comparison groups. As compared to the day of retrieval, 19, 11 and 6 miRNAs were differentially regulated more than 2 fold in the groups of no support, in the P support only, and in the P + E support respectively, 3–5 days after retrieval. During the peri-implantation period (3–5 days after retrieval) the expression of 33 and 6 miRNAs increased, while the expression of 3 and 0 miRNAs decreased, in the P alone and in the P + E group respectively as compared to the no steroid supplementation group.

**Conclusion:**

Luteal support following COS has a profound influence on miRNA profiles. Up or down regulation of miRNAs after P or P + E support suggest a role(s) of luteal support in the peri-implantation uterus in IVF cycles through the regulation of associated target genes.

## Background

MicroRNAs (miRNAs) are a class of single-stranded, non-coding small RNAs that regulate gene expression at the translational level and play fundamental roles in several biological processes, including cell differentiation, proliferation, development and apoptosis [1-3]. It is believed that mammalian miRNAs are responsible for the regulation of over 60% of all human genes [4]. Either by controlling mRNA degradation or by translational repression, miRNAs have emerged as key regulators of gene expression [5,6]. Each miRNA is predicated to have a broad range of target mRNAs and each mRNA may be regulated by multiple miRNAs [7,8].

The role of miRNAs in the female reproductive system and particularly in the endometrium has been the focus of several studies in recent years [9,10]. So far it has been established that miRNAs are indeed expressed in the human endometrium and they are also subjected to hormonal regulation [10,11]. Hawkins et al. were able to identify a number of miRNAs that were differentially regulated in endometriotic tissues as compared to normal endometrium [12]. The overall regulatory role of miRNAs in the pathophysiology of endometriosis has been reviewed extensively by Ohlsson Teaque *et al.*[13].

Ovarian stimulation protocols with gonadotropins have been invariably associated with luteal phase deficiency and poor implantation rates [14,15]. While the exact reasons for this phenomenon are still unclear, luteal phase support, given to improve endometrial characteristics and to facilitate the implantation process, has been a standard practice. Progesterone is a universally accepted agent for luteal phase support and can be administered orally, intramuscularly, or vaginally [16,17]. Estrogens in the form of 17β- estradiol or estradiol valerate have also been used for luteal phase support [18], although studies aimed to evaluate the concept of estrogen addition during the luteal phase have lead to inconclusive results [14,19] . It has been suggested that during ovarian stimulation for IVF, the endometrial receptivity starts to occur in mid luteal phase after oocyte retrieval [20]. Prior to, and during the implantation process, the expression of multiple endometrial genes and gene products is highly regulated [21-23]. The role of miRNAs in regulating cellular processes during the endometrial transition has recently attracted a great deal of attention [10,24-28]. For example, Kuokkanen *et al*. reported distinct miRNA gene expression signatures in the late proliferative and mid-secretory phase endometrial epithelium [24]. However, the effect of different types of luteal support in relation to endometrial miRNA profiles during the period of peri-implantation has not been described. In this study, we have investigated the impact of two commonly used luteal phase support protocols, progesterone alone and progesterone plus estrogen, on the expression profiles of 526 miRNAs in the human endometrium following ovarian stimulation with a gonadotropin/ GnRH antagonist protocol.

## Methods

### Oocyte donors and ovarian stimulation

The study was approved by the Johns Hopkins Hospital Institutional Review Board. Nine oocyte donors who enrolled in the Johns Hopkins oocyte donation program participated in the study. All donors were 21 to 31 years of age and underwent a standard screening protocol for oocyte donation, in accordance with the recommendations of the American Society for Reproductive Medicine [29]. The risks of the procedure were discussed in detail, with particular emphasis on the risks associated with the endometrial biopsy and the use of steroids during luteal phase, and written informed consents were obtained.

Study subjects underwent ovarian stimulation according to a gonadotropin / GnRH antagonist protocol as described previously [30]. Briefly, ovarian stimulation was initiated with gonadotropins on the second day of vaginal bleeding following discontinuation of oral contraceptive pills. On the 6^th^ day of stimulation, a daily subcutaneous evening dose of 0.25 mg ganirelix acetate (Schering-Plough Corp, West Orange, NJ, USA) was added. When at least three follicles reached a mean diameter of 18 mm, ovulation was triggered with a single dose of hCG (Profasi, 10,000 IU; Serono Inc. Rockland, MA, USA). Sonographically guided transvaginal oocyte retrieval was performed 34–36 hours after the hCG administration. The retrieved oocytes were used for IVF procedures and the resulting embryos were either transferred to matched recipients or cryopreserved for future use.

### Luteal-phase support and tissue collection

Endometrial biopsies on oocyte donors were performed using a Pipelle catheter (Unimar, Wilton, CT) on the day of oocyte retrieval and served as baseline (group I). At that time, the donors were randomized into three groups, with three subjects in each group. Group IIa received no luteal phase support after retrieval. Group IIb had luteal phase support with micronized progesterone (P) in the form of vaginal suppositories (200 mg every 6 h starting from the day after retrieval). Group IIc received a daily oral dose of 2 mg 17β-estradiol in addition to the micronized progesterone (P + E). Endometrial biopsies were obtained again 3–5 days (each of treatment groups contains 2 samples from day 3 and 1 sample from day 5) after retrieval. All specimens were stored in liquid nitrogen at −196°C immediately after the biopsy.

### RNA preparation and miRNA analysis

Total RNA was isolated and extracted from individual endometrial samples using the Trizol Reagent method (Invitrogen, Carlsbad, California 92008, cat. no. 15596–026). The quality of the RNA samples was assessed using an Agilent 2100 Bioanalyzer (Agilent Technologies, Palo Alto, CA). The integrity of miRNA was assessed by a miRNA specific RT-PCR using an ABI (Applied Biosystems; Foster City, CA) Taqman assay for U6 snRNA (AB Assay ID 001973). The results indicated an average Ct of 20.1 (SD 0.84) for all samples with a minimum Ct of 18.3 and maximum Ct of 22.

Illumina miRNA expression profiling (Catalog # MI-501-1001) was carried out according to manufacturer’s recommended protocols. Briefly 200ngs of total RNA for each sample was polyadenylated and converted to cDNA using a biotinylated oligo-dT primer with a universal PCR sequence at its 5’-end. Biotinylated cDNA was annealed to query oligos. Each query oligo consisted of a universal PCR priming site at the 5’end, an address sequence that complements a corresponding capture sequence on the array, and a microRNA-specific sequence at the 3’end. This mixture was bound to streptavidin-conjugated paramagnetic particles to select the cDNA/oligo complexes; second strand cDNA synthesis was completed by primer extension. All cDNA templates were amplified with a pair of common PCR primers. The primer on the strand complementary to the array was fluorescently labeled for subsequent hybridization to the arrays.

Validation of the selected miRNAs, shown to be regulated by Illumina miRNA microarray, was performed by RT-PCR. QRT-PCR was performed using the RT^2^ Profiler^TM^ Human miFinder miRNA PCR Array (MAH-001A) from SuperArray (SABiosciences, Gaithersburg, MD). RT^2^ Profiler™ PCR Arrays are designed for relative quantitative QRT-PCR based on SYBR Green detection and performed on a one sample/one plate 96-well format, using primers for a preset list of 88 most abundantly expressed and best characterized micro RNA sequences. In brief, miRNA was converted to cDNA via a universal tailing and reverse transcription reaction. CDNA volumes were adjusted to ~2.5 ml with SuperArray RT^2^ Real-Time SYBR Green/ROX PCR 2X Master Mix (PA-012) and 25 μl of cDNA mix was added to all wells. The PCR plate was sealed and spun at 1500 rpm X 4 min. Real time PCR was performed on an Applied Biosystem (Foster City, CA) 7300 Real Time PCR System. ABI instrument settings included setting reporter dye as “SYBR”, passive reference is “ROX”; Delete UNG Activation, and add Dissociation Stage.

To correlate differentially expressed miRNAs and their regulated genes, we used differentially regulated and selected miRNAs against an established miRNA database for predicted target genes (Sanger miRBase, v9.1, February 2007 release). MicroRNA data was also analyzed through the use of Ingenuity Pathway Analysis (IPA, Ingenuity® Systems, http://www.ingenuity.com). Pathway enrichments were calculated using the NIAID DAVID functional enrichment tool [31,32].

### Statistical analysis

Preliminary analysis of the scanned data was performed using Illumina BeadStudio software which returns single intensity data values/miRNA following the computation of a trimmed mean average for each probe type represented by a variable number of bead probes/gene on the array. Data was globally normalized by scaling each array to a common median value, and significant changes in gene expression between class pairs were calculated using the Student t-test. Significant gene lists were calculated by selecting genes which satisfied a significance threshold criteria of t-test *p*-values less than or equal to 0.05 and a fold change ± 2 or greater.

Relative miRNA expression derived from QRT-PCR was calculated by using the 2^-Ct^ method, in which Ct indicates cycle threshold, the fractional cycle number where the fluorescent signal reaches detection threshold [33]. The normalized ΔCt value of each sample is calculated using an endogenous control small molecular weight RNA (U6 snRNA). Fold change values are presented as average fold change = 2^-(average Ct)^ for genes in treated relative to control samples. The criteria of significance used for the RT-PCR results were the same as used for the Illumina miRNA arrays.

## Results

### Demographic characteristics

Demographic characteristics for all study participants were similar in all treatment groups. The mean age of the study participants was 24 years and mean body mass index was 21.3 ± 1.2 kg/m2. Overall, the baseline serum FSH, LH and E2 levels, the length of the stimulation , total amount of gonadotropins used, peak estradiol levels, and number of oocytes retrieved were comparable (P > 0.05) between the groups (Table 1).

**Table 1:** Group characteristics

**Characteristics**	**Group IIa (no support)**	**Group IIb (P support)**	**Group IIc (P + E support)**	**P**
*N*	3	3	3	
*Age (yr)*	25.7 ± 3.2	23.6 ± 0.8	22.8 ± 1.2	0.494
*BMI (kg/m*^*2*^*)*	23.3 ± 1.4	21.6 ± 1.8	20.2 ± 1.2	0.096
*Day 2 FSH (mIU/ml)*	4.5 ± 0.9	5.6 ± 1.1	4.0 ± 0.3	0.178
*Day 2 LH (mIU/ml)*	2.4 ± 0.8	4.0 ± 1.3	5.2 ± 0.2	0.507
*Day 2 E2 (pg/ml)*	36.7 ± 11.6	34.5 ± 12.5	20.5 ± 3.5	0.646
*Gonadotropins used (IU)*	2850 ± 525	2400 ± 645	2625 ± 675	0.387
*Peak E2 level (pg/ml)*	1928 ± 100.0	2514 ± 400	2625 ± 480	0.563
*Days of stimulation*	10.3 ± 1.1	9.3 ± 1.2	10.1 ± 0.7	0.588
*No. of oocytes*	14.5 ± 5	18.4 ± 3	16.0 ± 4	0.398

### MiRNA profiles and comparisons between groups

**Figure 1:**
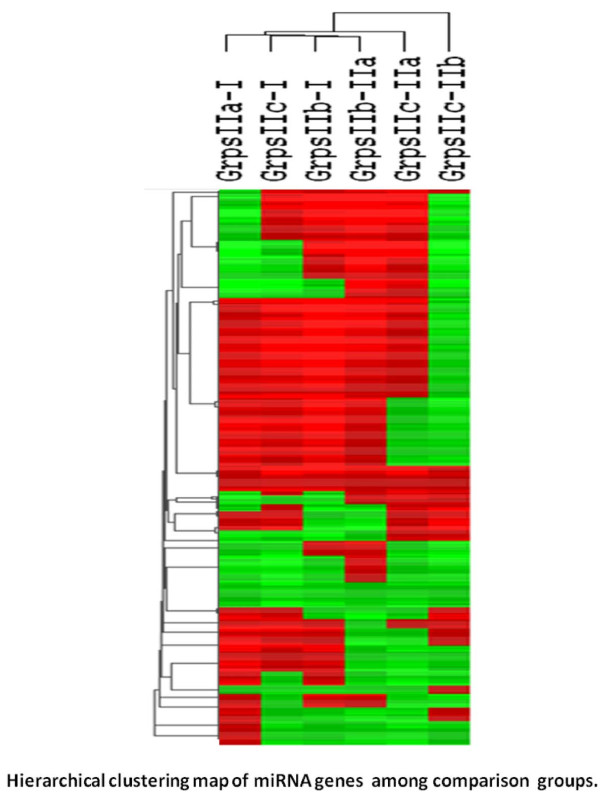
**Hierarchical clustering map of miRNA genes in all comparison groups: day 3–5 vs. day 0. (grpIIa-grpI = no luteal support vs. no luteal support; grpIIb-grpI = P support vs. no luteal support; grpIIc-grpI = P + E support vs. no luteal support) and day 3–5 vs. day 3–5 (grpIIb-grpIIa = P support vs. no support; grpIIc-grpI = P + E support vs. no support; grpIIc-grpIIb = P + E support vs. P support only).** Increased (red), decreased (green), and unchanged (yellow) miRNA levels from each transcript are indicated for each comparison group.

To establish endometrial miRNA profiles, we used a microarray platform consisting of 526 miRNA probes. Triplicates of each group samples were used, which proved that genes from same condition of samples are reproducible. Levels of miRNA expression are similar in the same sample groups including the samples from either day 3 or day5. The fluorescent intensity of each expressed transcript in each sample group was compared to the median fluorescence intensity of each transcript in the paired comparison group. Individual transcripts with increased (red) and decreased (green) miRNA abundance in the given comparisons were identified, as shown in the hierarchical clustering map in Figure 1. It is demonstrated that there is a high degree of overall concordance between and within treatments for later versus early luteal phase and, in particular a striking concordance, for hormone treated versus non-treated groups at days 3–5 after oocyte retrieval. Following global normalization, the mean expression value for each group was subjected to statistical analysis. A 2 fold change in the expression was arbitrarily selected as a cut-off level. Individual miRNAs that have shown a significant change in their expression (greater than 2fold and/or p < 0.05 between the comparison groups) are shown in an Additional file 1: Table S1 with a total of 248 miRNAs listed.

**Figure 2:**
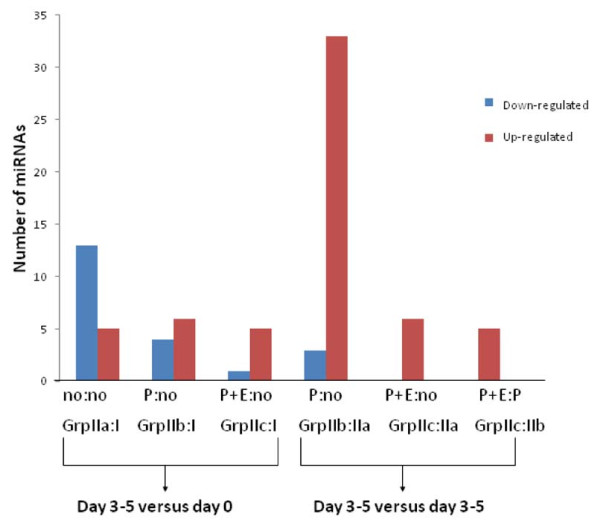
**Numbers of miRNA genes with more than 2 fold changes between comparison groups.** no = no steroid supplementation; P = progesterone support; P + E = progesterone + estrogen support.

Initially we compared miRNA expression in the endometrial samples obtained on the day of retrieval to those obtained 3–5 days later (Figure 2, the 3 comparison columns on the left). In the group with no luteal phase support, 14 miRNAs (HS_202.1, HS_209.1, HS_284.1, hsa-miR-202*:9.1, hsa-miR-346, hsa-miR-363*, hsa-miR-504, hsa-miR-569, hsa-miR-302d, hsa-miR-632, HS_17, HS_145.1, hsa-miR-133b, hsa-miR-144:9.1) were down-regulated and 5 miRNAs were up-regulated (HS_130, hsa-miR-876-5p, hsa-miR-876-3p, hsa-miR-122, hsa-miR-9) at greater than 2 fold changes. In the P alone group, 4 miRNAs (hsa-miR-144:9.1, hsa-miR-486-5p, HS_97, HS_203) were down regulated and 7 (HS_163, hsa-miR-614, hsa-miR-610, hsa-miR-559, hsa-miR-876-5p, HS_18, hsa-miR-876-3p) were up regulated, while in the P + E support group, 1 miRNA (hsa-miR-449a) was underexpressed and 5 (HS_276.1, hsa-miR-876-5p, HS_18, HS_111, hsa-miR-876-3p) were overexpressed .

Subsequently, we compared miRNA gene expression between the different treatment groups during mid-luteal phase at 3–5 days after retrieval, as shown in Figure 2, the 3 comparison columns on the right. In the progesterone support group an overexpression (more than 2 fold increase) was observed for 33 miRNAs (HS_149, HS_166.1, HS_175, HS_202.1, HS_209.1, HS_284.1, HS_41, hsa-miR-1468, hsa-miR-202*:9.1,hsa-miR-346, hsa-miR-504, hsa-miR-512-5p, hsa-miR-560:9.1, hsa-miR-563, hsa-miR-638, hsa-miR-663, hsa-miR-302d, hsa-miR-302b*, hsa-miR-632, hsa-miR-622, HS_17, HS_163, hsa-miR-518b, HS_108.1, hsa-miR-614, hsa-miR-610, HS_263.1, HS_30, hsa-miR-512-3p, HS_32, HS_282, HS_169, HS_145.1) and in the P + E support group for 6 miRNAs (HS_149, HS_276.1, HS_41, hsa-miR-563, HS_17, hsa-miR-144:9.1) as compared to the no steroid supplementation group. On the other hand, underexpression of 3 miRNAs (HS_176, HS_97, HS_203) were seen only in P support group. In the comparison between E + P and P supplementation groups, 5 miRNAs (hsa-miR-144:9.1, hsa-miR-486-5p, HS_176, HS_97, HS_203) were up-regulated and none were down regulated at greater than 2 fold levels.

### Venn diagram analysis of differentially expressed miRNA genes

**Figure 3:**
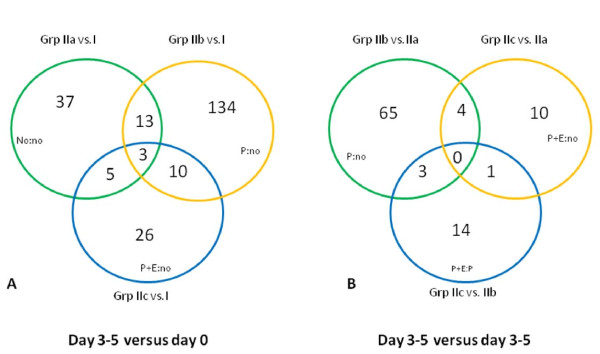
**Venn diagram illustrations of differentially expressed miRNA genes in six comparison groups.** Number of miRNA genes that were differentially expressed (p < 0.05) in the endometrium with and without luteal phase support as compared 3–5 days after oocyte retrieval versus day of retrieval (**A**) and at 3–5 days after oocyte retrieval (**B**) among groups. no = no steroid supplementation; P = progesterone supplementation; P + E = progesterone + estrogen supplementation.

A total of 216 miRNAs were differentially regulated (p < 0.05) between the study groups. MiRNAs with significant changes in common (shared miRNAs) between groups are shown in Figure 3. Panel A shows changes in miRNA expressions between day 3–5 and day of retrieval. Among the 3 comparison groups, 3 miRNAs (hsa-miR-876-3p, hsa-miR-155, and hsa-miR-503) were shared by all 3 groups and 5, 10 and 13 miRNAs respectively were shared in each pair of groups. Panel B compares groups on day 3–5 at all possible combinations. Group IIb vs. IIa and Group IIc vs. IIa shared 4 miRNAs (HS_241.1, hsa-miR-346, hsa-miR-503, and hsa-miR-99a); Group IIc vs. IIa and Group IIc vs. IIb shared 1 miRNA (hsa-miR-766) and Group IIb vs. IIa and Group IIc vs. IIb shared 3 miRNAs (hsa-miR-501-5p, hsa-miR-512-5p and hsa-miR-146a).

### Validation analysis

**Figure 4:**
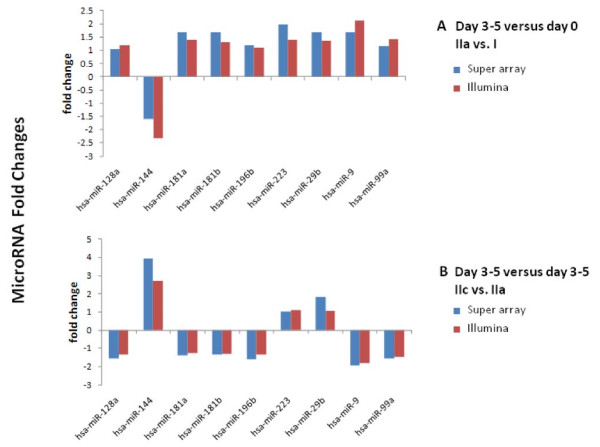
Validation results of the microarray findings for 9 miRNAs.

Array based RT-PCR with 88 miRNAs was used to validate our Illumina array expression findings. We were able to map 19 miRNAs between the two platforms. Of these, 14/19 demonstrated concordance at the level of the direction of regulation (increased or decreased) at a hypergeometric probability of p < 0.014. Nine representative miRNAs were selected for groups IIa vs. I and IIc vs. IIa as indicated in Figure 4. The trends for up-regulation and down- regulation of these miRNAs were consistent between the two array measurements.

### MiRNA and target genes

To explore the biological relationship between differentially expressed miRNAs and their regulated genes, we used differentially regulated (p < 0.05) miRNAs on day 3–5 after oocyte retrieval against an established miRNA database for predicted target genes (Sanger miRBase, v9.1, February 2007 release). Interestingly, there are large numbers of predicted target genes for a given miRNA per miRBase. We were able to identify nineteen miRNAs and their selected target genes in this defined study categories which are shown in Table 2.

**Table 2:** Selected miRNAs and gene targets (comparisons are at day3-5 after oocyte retrieval)

**miRNA**	**P vs. no**	**P + E vs. no**	**P + E vs. P**	**Predicted target genes**
*hsa-miR-335*	**↑↑**			IMP2,CD79B,WWP1,AP3S1,HOXD8,MAX,SP1,MAP2,MAK3,STARD7,CAP350,PANK2,SRPR,PPP6C,LASS5,ATP1B1
*hsa-miR-346*	**↑**	**↑**		IMP1,EIF3S1,BCL6,ABCC12,LIF,FSTL4,KGFLP2,KRAS,RAF7,FGF7,TMEM28,IGSF4B,PPP1R9B,COL2A1,HCG3, CALN1,HBP1,SF1
*hsa-miR-448*	**↑**			DOCK9,PPM2C,NTF3,CAP1,MAP2K6,ITM1,PRKAR2B,PAPPA,CDC2L6,CNTN4,IGF1R,SOCS5,CLK1,HOXA11,WWP1,FOXJ3,WDR22,MPPED2,ADD1,PRKA2
*hsa-miR-504*	**↑**			DCX,ATP1B4,IL1RAPL1,MNT,KLF13,PRKAR2A,IL16,LIF,FXR2,NRF1,CAMK2G,MMD,LOC284296,DND1,CNTFR, SORT1,NFIX
*hsa-miR-512-5p*	**↑↑**		**↓**	PIK3R1,CTNNB1,EMX2,SOX21,RIPK5,MBD6,SRPK, VNN3,ERP29,PHF15,FBXW11,LOC285074,MAP1A,CHD9
*hsa-miR-520 g*	**↑↑**			ETF1,CAMK2N1,NLK,TNFSF11,CNR1,EFTUD1,HMGB3,FBN2,ENC1,MARK4,TFEB,TNFSF12,PRKAR2A,TNKS1BP1,EIF4E,PPP3CA,IMP1,MAP3K14,TMTC2,TTN,GTF2IRD2,PTK2B,DNAJB5,TNRC6A,VEGF,EIF4G2,FOXO1A,MAP3K9
*hsa-miR-204*	**↑**			RUNX2,SOX4,NRBF2,MAP1LC3B,CDC42,ATP2B1,AKAP1,MAP3K3,CENTD1,IGF2R,NTRK2,TGFBR2,AP3M1,
				NEUROG1,P53CSV,TCF7L1,CDH2, CDC25B,TCF12,ELF2
*hsa-miR-369-3p*			**↓**	TCF8,PKIA,TLN1,CHD7,NCK2,CD2AP,CDC2L6,ELMOD1,CCNE2,FOXG1B,HOXB3,ADAMTS19,GIT2,ADAMTS3,TCF12, SRPK2,ADAMTS6,MAP2,ADAM10,FOXO1A,VEGFC
*hsa-miR-328*	**↑**			AK6,ITGA5,PRX,IGSF4C,MAX,SOX11,PTPN9,DPH2, HIST1H4D,USP37,VSIG4,RPP14,SF4,ULK2,FGD1,PLAG1
*hsa-miR-186*	**↑**			APLP2,ITGA6,RPS6KB1,CDC42,PRDM10,IGSF11,EFCBP1,TCF20,CAST,LMBR1,TMED2,TGFBR2,ICMT,IL2,CCNT2,HOXB8,PAK7,FOXD1,PTGES3,MAP3K2,VEGF,COL3A1, SRPK2,MAPKAP1,C16orf52,MAP2
*hsa-miR-517a*	**↑**			AMMECR1,ACACA,NPAS4,BSN,HNRPU,PTK2B,CDKN2A,CBLN2,RAPGEFL1,LOC201895,FOXJ3,PHF13,TMCC1
*hsa-miR-365*			**↓↓**	EIF4E3,MAP2K7,LAMP2,ENTPD7,PCNP,ADAMTS6, COL7A1,PPP5C,REV3L,PTGDR,KCNH2,RBM12,PKD2L2
*hsa-miR-221*	**↓↓**			CDC2L6,TIMP3,EIF4E3,NTF3,IMP2,HTLF,CDV3,NL,EIF5A2,NRK,PAK1,CDKN1C,FAT2,LIFR,TMCC1,MAP3K10,VGLL4,FAM13A1,TCF12,HOXC10,MAPK10,HMGCLL1,ADAM11,CD4,CTCF
*hsa-miR-495*	**↓↓**			MAPK6,CDK6,EML4,ILF3,PTK9,PRR7,HBEGF,HOMER1,MARK3,SP4,TGFB2,LHX2,HOXC6,PRKX,AP3M1,SOX9,GMFB,HMGCLL1,FOXO3A,EDG3,NKRF,HOXB9,TIMP2, IGSF4,CD164,TNFRSF21
*hsa-miR-146a*	**↓**		**↑**	FBXL10,IRAK1,TRAF6,CD79B,SP8,FLJ33814,SFRS6,NPAS4,CXorf22,EIF4G2,MMP16,USP3,KCTD15,SMAD4,LOC440944,SEC23IP,BCORL1,TM6SF2,DLGA1
*hsa-miR-99a*	**↓**	**↓**		EPDR1,FZD8,HS3ST2,EIF2C2,HS3ST3B1,FGFR3,BAZ2A,MBNL1,CYP26B1,KBTBD8,SMARCA5,FRAP1,ZZEF1,ICMT,C4orf16,ADCY1,MTMR3,CTDSPL,HOXA,RAVER2, INSM1,TRIB2,SLC44A1
*hsa-miR-181c,d*	**↑↑**			ETF1,COL16A1,NLK,TNFSF11,MAP3K3,MAP2K1,ITGA3,TCERG1,MAPT,MAPK1,MAP1B,CDH13,ITGB8,PCGF2,ADAMTS18,LMBRD2,MMP14,CD163,LIF,ADAMTS6, TNFRSF11B,CDC42BPA
*hsa-miR-200b*			**↓**	TCF8,NTF3,CYLN2,HMGB3,PRKAR2B,MPP5,GIT2,MAP4K3,FLJ21103,E2F3,CSNK1G3,MMD2,ZNF53, EIF5B,ERRFI1
*hsa-miR-196b*		**↓**		IMP1,CDYL,COL14A1,SSR1,IMP3,CDV3,CALM3,COL24A1,CDKN1B,ELF4,HOXC8,HMGA2,HOXA5,MAP4K3,PARP6,COL3A1,HOXA1,TNFSF12,COL1A2, HOXA7,HOXB6

**↑** or **↓**= up or down regulated, *p* < 0.05; **↑↑** or **↓↓**= up or down regulated, *p* < 0.01.

See website http://www.mirbase.org (http://microrna.sanger.ac.uk) regarding additional predicted target genes.

In order to further investigate the possible biological implications for those miRNAs which were cross validated by both QRT-PCR and Illumina array data (Figure 4), the relationship of these microRNAs and their known gene targets was evaluated using the IPA miRNA Target Filter software. This group of miRNAs is regulated between day 3–5 and day 0 and also at day 3–5 between P + E (Grps IIc) and no support (Grps IIa) groups. IPA was able to identify 7 out of the 9 miRNAs from Figure 4 (excluding hsa-miR-144, and hsa-mir-181b). The gene targets were identified for these miRNAs based upon the selection of the most stringent criteria requiring experimental observation of a given miRNA and its target. Gene targets were further filtered for known involvement in endocrine system disorders. The results of this analysis (Table 3) that shows pathway enrichments were calculated for the entire gene set. The findings of the analysis demonstrated a significant involvement of genes of extracellular matrix, cell proliferation, and response to steroid hormone stimulus between days 3–5 versus day 0 at no steroid support groups (Table 3, Grps IIa-I). Interestingly, this effect was almost completely abrogated by progesterone and estrogen treatment (Table 3, GrpsIIc-IIa) for genes of cellular proliferation and response to steroid hormones but not for extracellular matrix.

**Table 3:** Cross validated miRNAs and their selected target genes

**Symbol**	**GrpsIIa-I [ILL-FC]**	**GrpsIIc-IIa [ILL-FC]**	**Source**	**Symbol**	**Entrez gene name**	**Pathway (enrichment)**	**P Value**	**FDR**
*miR-223-3p (GUCAGUU)*	1.389	1.111	1	VIM	vimentin			
*miR-223-3p (GUCAGUU)*	1.389	1.111	1,2	RHOB	ras homolog family member B			
*miR-223-3p (GUCAGUU)*	1.389	1.111	1	IRS1	insulin receptor substrate 1			
*miR-29b-3p (AGCACCA)*	1.375	1.068	2,3	TUBB2A	tubulin, beta 2A class IIa			
*miR-29b-3p (AGCACCA)*	1.375	1.068	1,2,3,4	SPARC	secreted protein, acidic, cysteine-rich (osteonectin)	extracellular matrix	1.29E-07	4.44E-06
*miR-29b-3p (AGCACCA)*	1.375	1.068	1,2,3	PIK3R1	phosphoinositide-3-kinase, regulatory subunit 1 (alpha)			
*miR-29b-3p (AGCACCA)*	1.375	1.068	2,3	MYBL2	v-myb myeloblastosis viral oncogene homolog (avian)-like 2			
*miR-29b-3p (AGCACCA)*	1.375	1.068	1,2	COL5A3	collagen, type V, alpha 3	extracellular matrix	1.29E-07	4.44E-06
*miR-29b-3p (AGCACCA)*	1.375	1.068	32,3	COL5A2	collagen, type V, alpha 2	extracellular matrix	1.29E-07	4.44E-06
*miR-29b-3p (AGCACCA)*	1.375	1.068	1,2,4	COL4A1	collagen, type IV, alpha 1	extracellular matrix	1.29E-07	4.44E-06
*miR-29b-3p (AGCACCA)*	1.375	1.068	1,2,3,4	COL1A2	collagen, type I, alpha 2	extracellular matrix	1.29E-07	4.44E-06
*miR-29b-3p (AGCACCA)*	1.375	1.068	1,2,4	COL15A1	collagen, type XV, alpha 1	extracellular matrix	1.29E-07	4.44E-06
*miR-9-5p (CUUUGGU)*	2.104	−1.802	1	NFKB1	nuclear factor of kappa light polypeptide gene enhancer in B-cells 1			
*miR-9-5p (CUUUGGU)*	2.104	−1.802	1,2	FOXO1	forkhead box O1			
*miR-9-5p (CUUUGGU)*	2.104	−1.802	1,2	FOXG1	forkhead box G1	positive regulation of cell proliferation	1.21E-08	1.67E-06
*miR-9-5p (CUUUGGU)*	2.104	−1.802	1,2,3	CDH1	cadherin 1, type 1, E-cadherin (epithelial)			
*miR-181a-5p (ACAUUCA)*	1.376	−1.24	1,4	TRA@	T cell receptor alpha locus			
*miR-181a-5p (ACAUUCA)*	1.376	−1.24	1,2	TIMP3	TIMP metallopeptidase inhibitor 3			
*miR-181a-5p (ACAUUCA)*	1.376	−1.24	1,2	NOTCH4	notch 4	positive regulation of cell proliferation	1.21E-08	1.67E-06
*miR-181a-5p (ACAUUCA)*	1.376	−1.24	1,2	**KRAS**	v-Ki-ras2 Kirsten rat sarcoma viral oncogene homolog	response to steroid hormone stimulus	6.24E-07	4.65E-05
*miR-181a-5p (ACAUUCA)*	1.376	−1.24	1,2,4	HOXA11	homeobox A11			
*miR-181a-5p (ACAUUCA)*	1.376	−1.24	1,2	GATA6	GATA binding protein 6			
*miR-181a-5p (ACAUUCA)*	1.376	−1.24	1,2	ESR1	estrogen receptor 1	response to steroid hormone stimulus	6.24E-07	4.65E-05
*miR-181a-5p (ACAUUCA)*	1.376	−1.24	1,2	CDKN1B	cyclin-dependent kinase inhibitor 1B (p27, Kip1)	positive regulation of cell proliferation	1.21E-08	1.67E-06
*miR-181a-5p (ACAUUCA)*	1.376	−1.24	1,2,3,4	**BCL2**	B-cell CLL/lymphoma 2	response to steroid hormone stimulus	6.24E-07	4.65E-05
*miR-196a-5p (AGGUAGU)*	1.092	−1.342	1	IKBKB	inhibitor of kappa light polypeptide gene enhancer in B-cells, kinase beta			
*miR-196a-5p (AGGUAGU)*	1.092	−1.342	1,2,4	HOXC8	homeobox C8			
*miR-196a-5p (AGGUAGU)*	1.092	−1.342	1,3	ANXA1	annexin A1			
*miR-99a-5p (ACCCGUA)*	1.427	−1.48	1,2,3	MTOR	mechanistic target of rapamycin (serine/threonine kinase)	positive regulation of cell proliferation	1.21E-08	1.67E-06
*miR-99a-5p (ACCCGUA)*	1.427	−1.48	1,2,3	IGF1R	insulin-like growth factor 1 receptor	positive regulation of cell proliferation	1.21E-08	1.67E-06
*miR-99a-5p (ACCCGUA)*	1.427	−1.48	1,2,3	FGFR3	fibroblast growth factor receptor 3	positive regulation of cell proliferation	1.21E-08	1.67E-06
*miR-128 (CACAGUG)*	1.184	−1.345	1,2	TXNIP	thioredoxin interacting protein	response to steroid hormone stimulus	6.24E-07	4.65E-05
*miR-128 (CACAGUG)*	1.184	−1.345	2,3	**TGFBR1**	transforming growth factor, beta receptor 1	response to steroid hormone stimulus	6.24E-07	4.65E-05
*miR-128 (CACAGUG)*	1.184	−1.345	1,2	LDLR	low density lipoprotein receptor	response to steroid hormone stimulus	6.24E-07	4.65E-05
*miR-128 (CACAGUG)*	1.184	−1.345	1,2	E2F3	E2F transcription factor 3	positive regulation of cell proliferation	1.21E-08	1.67E-06
*miR-128 (CACAGUG)*	1.184	−1.345	1,2	ADORA2B	adenosine A2b receptor			

Ingenuity Pathway Analysis (Ingenuity® Systems, http://www.ingenuity.com). MiRNA Target Filter was applied using the strictest criteria (experimentally observed microRNA/gene targets only) filtered for genes previously identified for involvement in endocrine system disorders. Each of the seven miRNAs has multiple gene targets. Fold changes between groups as determined by Illumina miRNA array measurements are shown. Ingenuity target identifications are generated from multiple databases (Source). Pathway enrichments were calculated for the entire gene set using the NIAID DAVID functional enrichment tool [31,32]. Genes that feature in both the cellular proliferation and the steroid hormone pathways are in bold (KRAS, BCL2, TGFBR1).

[ILL-FC] = Illumina array-fold change; Source = 1.miRecords, 2.TargetScan Human, 3.Ingenuity Expert Findings, 4.TarBase; FDR = False Discover Rate.

## Discussion

In the past few years, the field of miRNA research has evolved rapidly. Various studies have provided strong evidence for the widespread expression and the regulatory functions of miRNAs on gene expression under either physiologic or pathologic conditions. MicroRNAs have now been recognized as key players in the process of cell proliferation and differentiation. Global analysis of miRNAs in human tissues have showed that, in addition to the brain, the uterus, the cervix, and the ovaries have the highest restricted enrichment in individual miRNAs [34]. The identification of miRNA as well as the functional analysis of individual expressed miRNA in the uterus has shed light onto the cycling changes that occur in response to steroids and during pregnancy. The impact of the ovarian steroids on miRNA expression and regulation in the uterus has been evidenced by the fact that treatment with 17β estradiol or RU-486 resulted in differential regulation of miRNAs in the myometrium and leiomyomas [35].

In the present study, we have examined 526 different miRNAs in the human endometrium following COS and identified a rich number of miRNAs with at least 2 fold changes in the level of expression during the luteal phase (Figure 2, Additional file 1: Table S1). Statistical analysis identified that the changes were significant (p < 0.05) for 216 of miRNAs ( Additional file 1: Table S1). These changes were observed not only in the within the group analysis at different times during luteal phase (comparison between day 0 and day 3–5) but also in the analysis between groups at the same time frame (comparison between the treatment groups at day 3–5). As demonstrated in Figure 1and Figure 2, there was a substantial increase in miRNA expression in the groups treated with progesterone alone as compared to the no supplementation group. In genome-wide identification of endometrial miRNA in natural and stimulated cycles reported by Sha *et al.*[36], 22 miRNAs were significantly dysregulated on the day of hCG + 7 in stimulated cycles as compared with day of LH + 7 in natural cycles. Among those, 11 miRNAs exhibited putative estrogen response elements or progesterone response elements in the promoters. In a study of examining gene expression profile in natural cycle and stimulated cycles during luteal phase (LH + 2 or 7; hCG + 2 or 7), Haouzi *et al.*[37] demonstrated that COS regimens altered endometrial receptivity in comparison with natural cycle. These and our studies indicate that ovarian stimulation or altered steroid hormone levels may affect miRNA profiles, consequently, affect endometrial receptivity. Furthermore, we found that the addition of estradiol in the regimen resulted in a significant attenuation of effect of progestone (Figure 1, Figure 2) on the level of miRNA expression. These findings support the notion that the well known anti-proliferative effect of progesterone on the endometrium could be possibly exerted by a localized increase in miRNA expression. The addition of estradiol at the same time could reverse this effect partially by attenuating this increase. Whether this effect is directly or indirectly associated with ovarian stimulation or the type of drug delivery for luteal support (estradiol was administered orally whereas progesterone was administered vaginally in this study) requires further investigation.

By microarray, Northen blot and in situ hybridization, Hu *et al*. [38] was able to identify eight specific miRNAs that were significantly up-regulated at implantation sites. Chakrabarty et al. have showed in the mouse uterus, that two specific miRNAs, the mmu-miR-101a and the mmu-miR-199a*, were differentially expressed during implantation in coordination with the expression of cyclooxygenase-2(Cox-2), a gene critical for implantation [39]. Studies on temporal and spatial regulation of miRNAs in the rat uterus, during embryo implantation, have identified the let-7a and mir-320 specifically in the uterine endometrium with higher expression level on gestation day 6–7 [26,27]. These evidences and our findings of differential expression of miRNAs in the peri-implantation period with and without luteal phase support suggest role(s) of miRNAs during the remodeling process of endometrium in association with implantation.

Neo-angiogenesis is a pivotal process in reproductive function where it regulates endometrial regeneration, corpus luteum formation and finally placentation. The regulatory function of miRNAs in the process of neo-angiogenesis has been illustrated in several in vitro and in vivo models [9]. For example, the role of miRNAs in the neo-angiogenesis has been reported in experiments with Dicerex Â½ mouse embryos (altered function of Dicer required for miRNA processing) which suffer from defective angiogenesis, due to disruption in the expression of vascular endothelial growth factor (VEGF) as well as to its receptor flt-1 [40]. We have noticed in our study that several miRNAs including miR-520 g, miR-369-3p, and miR-186 (Table 2), with VEGF as predicted target gene, were differentially regulated during the peri-implantation period. More specifically there was a significant increase in the expression of miR-520 g in the group that received only progesterone as compared to the other groups. In contrast, in the same group, there was a pronounced suppression of miR-221, which is known to regulate endothelial nitric oxide synthase, one of the key regulators of endothelial biology and angiogenesis [41]. Whereas our findings support the regulatory effect of miRNAs in the process of neo-angiogenesis, the precise impact of this action remains obscure. Individual targets of specific miRNAs responsible for the phenotypes have been proposed in experimental settings, although it is likely that many miRNAs function through cooperative regulation of multiple mRNAs [7]. Indeed, Revel et al. evaluated the expression of miRNAs in the secretory endometrium of repeated implantation failure patients and identified 13 miRNAs were differentially expressed (10 were overexpressed and 3 were underexpressed), which putatively regulated the expression of 3800 genes.

In addition, in this study, based on the most stringent criteria requiring experimental observations, IPA miRNA Target analysis for cross validated microRNAs identified 7 out of 9 miRNAs and their gene targets which were further subjected for pathway analysis. The results revealed significant involvement of genes of extracellular matrix, cell proliferation, and response to steroid hormone stimulus from day 0 to day 3–5 after oocyte retrieval in a group with no steroid support (Table 3). Conversely, this effect was almost completely abolished by supplementation of progesterone and estrogen (Table 3, GrpsIIc-IIa) for genes of cellular proliferation and response to steroid hormones bur not for genes of extracellular matrix.

Under the influence of the ovarian steroids the human endometrium undergoes cyclic changes. Estradiol promotes epithelial cell proliferation, while progesterone inhibits this estrogen-induced effect, promotes differentiation, and has decidualizing effects on endometrial stroma later in the secretary phase. We hypothesize that ovarian steroids may regulate multiple genes related to the uterine tissue remodeling and endometrial receptivity, at least in part, through modulating miRNA expression profiles.

We realize that there are several limitations in this study. The relatively small sample size due to limited number of donors that have agreed to participate could represent one of those. Unfortunately due to the design of our experiment it was extremely difficult to obtain more specimens. Furthermore, due to the fact that the same women were biopsied twice during the same COS cycle the first biopsy may induce gene expression differences that are likely to be reflected in the miRNA expression profile of the second biopsy. Additional group(s) with only one biopsy for each subject for a given group and given day of biopsy would provide another layer of control to strengthen the findings in this study. On the other hand, the limited sample size also reflects the difficulty in obtaining these samples. In addition, although group II contains 3 samples in each sub-group, there are 2 samples from day 3 and 1 sample from day 5 which may potentially affect miRNA profiles. However, after normalization and careful comparison, samples from day 3 and day 5 showed similar expression level on miRNAs profile in the same treatment group. Since day 3–5 are all in mid-secretory period of the cycle, we combined day 3 and day5 samples as one stage of the luteal phase for analysis.

Despite these limitations nevertheless, our array-based global miRNA profiling describes, for the first time, the miRNA expression profile in the human endometrium during the luteal phase following COS for IVF and luteal support with steroid supplementation. We have shown that this profile is under considerable influence by ovarian steroids, even though the molecular mechanism of this interaction still remains unclear. Importantly, several miRNAs found to have enriched or depleted transcript load during the luteal phase may have specific roles in the control of endometrial receptivity. Further studies are necessary to create a detailed expression profile for these miRNAs in relation to their target genes in the endometrium throughout the natural cycle as well as the stimulated cycle for IVF. We plan to further investigate several significantly regulated miRNAs and associated target gene pathways in relation to endometrial receptivity and implantation. Functional study will also be designed to link the imperative miRNAs in potential clinical applications.

## Conclusions

The array-based study presented here has revealed several findings: 1) there is an expression of a unique set of miRNAs in the endometium following controlled ovarian stimulation; 2) the level of expression for these miRNAs undergoes significant changes during the peri-implantation period; 3) the expression is influenced by ovarian steroids; 4) expression of miRNAs may be associated with target genes and gene pathways. The miRNAs found to have enriched or depleted transcript load during the luteal phase may have specific roles in the control of endometrial receptivity during the peri- implantation period through regulation of their target genes. Further studies are necessary to create a detailed expression profile for these miRNAs as well as their associated target genes throughout the natural cycle and the stimulated cycle for IVF in the endometrium. Studies for specifically regulated miRNAs and their target genes as well specific gene pathways in relation to endometrial receptivity and implantation are also proposed.

## Abbreviations

miRNA, MicroRNAs; COS, Controlled ovarian stimulation; GnRH, Gonadotropic releasing hormones; IVF, In vitro fertilization; hCG, Human chorionic gonadotropin; LH, Luteinizing hormone; FSH, Follicle-stimulating hormone; P, Progesterone; P + E, Progesterone plus 17-beta-estradiol; IPA, Ingenuity pathway analysis.

## Competing interests

The authors declare that they have no competing interests.

## Authors’ contributions

YZ, NV designed the protocol, developed the study, managed specimens, and drafted the manuscript. HZ provided valuable suggestions for the study and actively involved in manuscript preparation. NN participated in data analysis and manuscript preparation. CC, JF carried out microarray analysis, performed the statistical analysis, and contributed in manuscript preparation. All authors read and approved the final manuscript.

## Supplementary Material

Additional file 1 ** Table S1.**MiRNA with greater than 2 fold changes and/or significantly regulated between comparison groups. Click here for file

## References

[B1] Bueno MJ de Castro Pérez I Malumbres M Control of cell proliferation pathways by microRNAs Cell Cycle 2008 20 3143 3148 18843198 10.4161/cc.7.20.6833

[B2] Engels BM Hutvagner G Principles and effects of microRNA-mediated post-transcriptional gene regulation Oncogene 2006 25 6163 6169 10.1038/sj.onc.1209909 17028595

[B3] Jovanovic M Hengartner MO miRNAs and apoptosis: RNAs to die for Oncogene 2006 25 6176 6187 10.1038/sj.onc.1209912 17028597

[B4] Friedman RC Farh KK Burge CB Bartel DP Most mammalian mRNAs are conserved targets of microRNAs Genome Res 2009 19 92 105 18955434 10.1101/gr.082701.108 PMC2612969

[B5] Ambros V Chen X The regulation of genes and genomes by small RNAs Development 2007 134 1635 1641 10.1242/dev.002006 17409118

[B6] Bartel DP MicroRNAs: genomics, biogenesis, mechanism, and function Cell 2004 116 281 297 10.1016/S0092-8674(04)00045-5 14744438

[B7] Lim LP Lau NC Garrett-Engele P Grimson A Schelter JM Castle J Bartel DP Linsley PS Johnson JM Microarray analysis shows that some microRNAs downregulate large numbers of target mRNAs Nature 2005 433 769 773 10.1038/nature03315 15685193

[B8] Revel A Achache H Stevens J Smith Y Reich R MicroRNAs are associated with human embryo implantation defects Hum Reprod 2011 26 2830 2840 10.1093/humrep/der255 21849299

[B9] Pan Q Luo X Toloubeydokhti T Chegini N The expression profile of micro-RNA in endometrium and endometriosis and the influence of ovarian steroids on their expression Mol Hum Reprod 2007 13 11 797 806 10.1093/molehr/gam063 17766684

[B10] Pan Q Chegini N MicroRNA signature and regulatory functions in the endometrium during normal and disease states Semin Reprod Med 2008 26 479 493 10.1055/s-0028-1096128 18951330 PMC2728121

[B11] Ohlsson Teague EM Van der Hoek KH Van der Hoek MB Perry N Wagaarachchi P Robertson SA Print CG Hull LM MicroRNA-regulated pathways associated with endometriosis Mol Endocrinol 2009 23 265 275 19074548 10.1210/me.2008-0387 PMC5419313

[B12] Hawkins SM Creighton CJ Han DY Zariff A Anderson ML Gunaratne PH Matzuk MM Functional MicroRNA involved in endometriosis Mol Endocrinol 2011 25 821 832 10.1210/me.2010-0371 21436257 PMC3082329

[B13] Ohlsson Teague EM Print CG Hull ML The role of microRNAs in endometriosis and associated reproductive conditions Hum Reprod Update 2010 16 142 165 10.1093/humupd/dmp034 19773286

[B14] Kovalevsky G Patrizio P High rates of embryo wastage with use of assisted reproductive technology: a look at the trends between 1995 and 2001 in the United States Fertil Steril 2005 84 325 330 10.1016/j.fertnstert.2005.04.020 16084872

[B15] Tavaniotou A Albano C Smitz J Devroey P Comparison of LH concentrations in the early and mid-luteal phase in IVF cycles after treatment with HMG alone or in association with the GnRH antagonist Cetrorelix Hum Reprod 2001 16 663 667 10.1093/humrep/16.4.663 11278214

[B16] Pabuccu R Akar ME Luteal phase support in assisted reproductive technology Curr Opin Obstet Gynecol 2005 17 277 281 10.1097/01.gco.0000169105.62257.e3 15870562

[B17] Posaci C Smitz J Camus M Osmanagaoglu K Devroey P Progesterone for the luteal support of assisted reproductive technologies: clinical options Hum Reprod Suppl 2000 1 129 148 10.1093/humrep/15.suppl_1.129 10928425

[B18] Pritts EA Atwood AK Luteal phase support in infertility treatment: a meta-analysis of the randomized trials Hum Reprod 2002 17 2287 2299 10.1093/humrep/17.9.2287 12202415

[B19] Kolibianakis EM Venetis CA Papanikolaou EG Diedrich K Tarlatzis BC Griesinger G Estrogen addition to progesterone for luteal phase support in cycles stimulated with GnRH analogues and gonadotrophins for IVF: a systematic review and meta-analysis Hum Reprod 2008 23 1346 1354 10.1093/humrep/den115 18408017

[B20] Navot D Veeck LL Scott RT Lui HC Droesch K Rosenwaks Z The window of embryo transfer and efficiency of human conception in vitro Ferti Steril 1991 55 114 118 10.1016/s0015-0282(16)54069-2 1986951

[B21] Zhao Y Garcia J Kolp L Cheadle C Rodriguez A Vlahos NF The impact of luteal phase support on gene expression of extracellular matrix protein and adhesion molecules in the human endometrium during the window of implantation following controlled ovarian stimulation with a GnRH antagonist protocol Fertil Steril 2010 94 2264 2271 10.1016/j.fertnstert.2010.01.068 20226447

[B22] Liu Y Lee KF Ng EH Yeung WS Ho PC Gene expression profiling of human peri-implantation endometria between natural and stimulated cycles Fertil Steril 2008 90 2152 2164 10.1016/j.fertnstert.2007.10.020 18191855

[B23] Reddy KV Mangale SS Integrin receptors: the dynamic modulators of endometrial function Tissue Cell 2003 35 260 273 10.1016/S0040-8166(03)00039-9 12921709

[B24] Kuokkanen S Chen B Ojalvo L Benard L Santoro N Pollard JW Genomic profiling of microRNAs and messenger RNAs reveals hormonal regulation in microRNA expression in human endometrium Biol Reprod 2010 82 791 801 10.1095/biolreprod.109.081059 19864316 PMC2842492

[B25] Qian K Hu L Chen H Li H Liu N Li Y Ai J Zhu G Tang Z Zhang H Hsa-miR-222 is involved in differentiation of endometrial stromal cells in vitro Endocrinology 2009 150 4734 4743 10.1210/en.2008-1629 19589872

[B26] Xia HF Jin XH Song PP Cui Y Liu CM Ma X Temporal and spatial regulation of miR-320 in the uterus during embryo implantation in the Rat Int J Mol Sci 2010 11 719 730 10.3390/ijms11020719 20386663 PMC2852863

[B27] Xia HF Jin XH Song PP Cui Y Liu CM Ma X Temporal and spatial regulation of Let-7a in the uterus during embryo implantation in the Rat J Reprod Dev 2010 56 73 78 10.1262/jrd.09-088K 19881221

[B28] Altmäe S Martinez-Conejero JA Esteban FJ Ruiz-Alonso M Stavreus-Evers A Horcajadas JA Salumets A MicroRNAs miR-30b, miR-30d, and miR-494 Regulate Human Endometrial Receptivity Reprod Sci 2012 [Epub ahead of print] 10.1177/1933719112453507 PMC4077381 22902743

[B29] American Society for Reproductive Medicine Guidelines for oocyte donation Fertil Steril 2002 77 Suppl 5 S6 S8 10.1016/s0015-0282(02)03182-5 12069860

[B30] Vlahos NF Lipari CW Bankowski B Lai TH King JA Shih IM Fragakis K Zhao Y Effect of luteal-phase support on endometrial L-selectin ligand expression after recombinant follicle-stimulating hormone and ganirelix acetate for in vitro fertilization J Clin Endocrinol Metab 2006 91 4043 4049 10.1210/jc.2006-0520 16868054

[B31] Huang DW Sherman BT Lempicki RA Systematic and integrative analysis of large gene lists using DAVID Bioinformatics Resources Nature Protoc 2009 4 44 57 19131956 10.1038/nprot.2008.211

[B32] Huang DW Sherman BT Lempicki RA Bioinformatics enrichment tools: paths toward the comprehensive functional analysis of large gene lists Nucleic Acids Re 2009 37 1 13 10.1093/nar/gkn923 PMC2615629 19033363

[B33] Livak KJ Schmittgen TD Analysis of relative gene expression data using real-time quantitative PCR and the 2(−Delta Delta C(T)) Methods 2001 25 402 408 10.1006/meth.2001.1262 11846609

[B34] Shingara J Keiger K Shelton J Laosinchai-Wolf W Powers P Conrad R An optimized isolation and labeling platform for accurate microRNA expression profiling RNA 2005 11 1461 1470 10.1261/rna.2610405 16043497 PMC1370829

[B35] Pan Q Luo X Chegini N Differential expression of microRNAs in myometrium and leiomyomas and regulation by ovarian steroids J Cell Mol Med 2008 12 227 240 18182067 10.1111/j.1582-4934.2007.00207.x PMC2730932

[B36] Sha AG Liu JL Jiang XM Ren JZ Ma CH Lei W Su RW Yang ZM Genome-wide identification of micro-ribonucleic acids associated with human endometrial receptivity in natural and stimulated cycles by deep sequencing Fertil Steril 2011 96 150 155 10.1016/j.fertnstert.2011.04.072 21601191

[B37] Haouzi D Assou S Mahmoud K Tondeur S Rème T Hedon B De Vos J Hamamah S Gene expression profile of human endometrial receptivity: comparison between natural and stimulated cycles for the same patients Hum Reprod 2009 24 1436 1445 10.1093/humrep/dep039 19246470 PMC2871799

[B38] Hu S Ren G Liu JL Zhao ZA Yu YS Su RW Ma XH Ni H Lei W Yang ZM MicroRNA Expression and Regulation in Mouse Uterus during Embryo Implantation J Bio Chem 2008 283 23473 23484 10.1074/jbc.M800406200 18556655

[B39] Chakrabarty A Tranguch S Daikoku T Jensen K Furneaux H Dey S MicroRNA Regulation of cyclooxygenase-2 during embryo implantation PNAS 2007 104 15144 15149 10.1073/pnas.0705917104 17848513 PMC1986627

[B40] Yang WJ Yang DD Na S Sandusky GE Zhang Q Zhao G Dicer is required for embryonic angiogenesis during mouse development J Biol Chem 2005 280 9330 9335 15613470 10.1074/jbc.M413394200

[B41] Suárez Y Fernández-Hernando C Pober JS Sessa WC Dicer dependent microRNAs regulate gene expression and functions in human endothelial cells Circ Res 2007 100 1164 1173 10.1161/01.RES.0000265065.26744.17 17379831

