# Supplementary material for: Effect of luteal-phase support on endometrial microRNA expression following controlled ovarian stimulation
Source: Reprod Biol Endocrinol. 2012 Sep 6;10:72. doi: 10.1186/1477-7827-10-72 (PMC3462109; doi:10.1186/1477-7827-10-72)
Supplement: Additional file 1 — Table S1.MiRNA with greater than 2 fold changes and/or significantly regulated between comparison groups. [file 1477-7827-10-72-S1.docx]

Table 1. MiRNA with greater than 2 fold changes and/or significantly regulated between comparison groups

|  | **Day 3-5 vs. Day 0** | | | **Day 3-5 vs. Day 3-5** | | |
| --- | --- | --- | --- | --- | --- | --- |
| ILMN_GENE | IIa vs.I | IIb vs.I | IIc vs.I | IIb vs.IIa | IIc vs.IIa | IIc vs.IIb |
|  | (no vs. no) | (P vs. no) | (P+E vs. no) | (P vs. no) | (P+E vs. no) | (P+E vs P) |
| HS_149 |  | 1.64 ^a^ |  | 2.77 ^b^ | 2.18 |  |
| HS_166.1 |  |  |  | 2.17 ^a^ |  |  |
| HS_175 |  | 1.55^b^ |  | 2.17 ^b^ |  |  |
| HS_202.1 | -2.01 |  |  | 3.47 ^a^ |  |  |
| HS_209.1 | -2.19 |  |  | 2.35 ^a^ |  |  |
| HS_22.1 |  |  |  | 1.83 ^a^ |  |  |
| HS_241.1 |  |  |  | 1.62 ^a^ | 1.41 ^a^ |  |
| HS_257 |  | 1.53 ^a^ |  | 1.55 ^a^ |  |  |
| HS_276.1 |  | 1.88 ^a^ | 2.55 | 1.75 ^a^ | 2.37 |  |
| HS_284.1 | -2.37^a^ |  |  | 2.56 ^b^ |  |  |
| HS_41 |  | 1.73 ^a^ |  | 3.07 ^a^ | 2.48 |  |
| hsa-miR-1468 |  | 1.72 ^b^ |  | 2.11 ^a^ |  |  |
| hsa-miR-181c |  | 1.56 ^a^ |  | 1.70 ^b^ |  |  |
| hsa-miR-202*:9.1 | -2.22 ^a^ |  |  | 2.76 ^b^ |  |  |
| hsa-miR-296-3p |  |  |  | 1.51 ^a^ |  |  |
| hsa-miR-335 | -1.66 ^a^ |  |  | 1.74 ^b^ |  |  |
| hsa-miR-346 | -2.44 |  |  | 3.14 ^a^ | 1.81 ^a^ |  |
| hsa-miR-363* | -2.44 |  |  | 1.95 ^a^ |  |  |
| hsa-miR-425* | -1.37 ^a^ | 1.32 ^b^ |  | 1.82 ^b^ |  |  |
| hsa-miR-448 |  | 1.68 ^b^ |  | 1.63 ^a^ |  |  |
| hsa-miR-491-5p | -1.54 ^a^ |  |  | 1.67 ^a^ |  |  |
| hsa-miR-501-5p |  |  |  | 1.57 ^a^ |  | -1.61 ^a^ |
| hsa-miR-504 | -2.03 |  |  | 2.88 ^a^ |  |  |
| hsa-miR-512-5p |  |  |  | 2.12 ^b^ |  | -1.68 ^a^ |
| hsa-miR-519b-3p |  |  |  | 1.79 ^a^ |  |  |
| hsa-miR-520g |  | 1.55 ^b^ |  | 1.71 ^b^ |  |  |
| hsa-miR-560:9.1 |  |  |  | 2.51 ^b^ |  |  |
| hsa-miR-563 |  | 1.42 ^a^ |  | 2.72 ^a^ | 2.28 |  |
| hsa-miR-569 | -2.23 |  |  | 1.99 ^a^ |  |  |
| hsa-miR-584 |  | 1.39 ^a^ |  | 1.94 ^a^ |  |  |
| hsa-miR-595 |  | 1.76 ^a^ |  | 1.77 ^a^ |  |  |
| hsa-miR-638 |  |  |  | 2.05 ^a^ |  |  |
| hsa-miR-663 |  |  |  | 2.08 ^a^ |  |  |
| hsa-miR-302d | -2.14 |  |  | 3.61 |  |  |
| hsa-miR-302b* |  |  |  | 3.52 |  |  |
| hsa-miR-632 | -2.12 |  |  | 3.37 |  |  |
| hsa-miR-622 |  |  |  | 3.37 |  |  |
| HS_17 | -2.05 |  |  | 3.20 | 2.41 |  |
| HS_163 |  | 2.14 |  | 2.59 |  |  |
| hsa-miR-518b |  |  |  | 2.53 |  |  |
| HS_108.1 |  |  |  | 2.44 |  |  |
| hsa-miR-614 |  | 2.28 |  | 2.42 |  |  |
| hsa-miR-610 |  | 2.21 |  | 2.40 |  |  |
| HS_263.1 |  |  |  | 2.24 |  |  |
| HS_30 |  |  |  | 2.15 |  |  |
| hsa-miR-512-3p |  |  |  | 2.11 |  |  |
| HS_32 |  |  |  | 2.11 |  |  |
| HS_282 |  |  |  | 2.08 |  |  |
| HS_169 |  | 1.97 ^a^ |  | 2.02 |  |  |
| HS_145.1 | -2.31 |  |  | 2.01 |  |  |
| HS_117 |  |  |  |  |  |  |
| HS_188 |  | 1.90 ^a^ |  |  |  |  |
| hsa-miR-559 |  | 2.65 |  |  |  |  |
| HS_130 | 2.21 |  |  |  |  |  |
| hsa-miR-891a |  | 1.91 ^a^ |  |  |  |  |
| HS_8 |  | 1.73 ^b^ |  |  |  |  |
| hsa-miR-566 |  | 1.60 ^a^ |  |  |  |  |
| hsa-miR-133b | -2.09 ^a^ |  |  |  |  |  |
| HS_260 |  |  |  |  |  |  |
| hsa-miR-876-5p | 2.26 | 3.50 ^a^ | 3.02 ^b^ |  |  |  |
| HS_31.1 |  | 1.93 ^b^ |  |  |  |  |
| HS_18 |  | 2.15 ^a^ | 2.03 |  |  |  |
| hsa-miR-211 |  | 1.62 ^a^ |  |  |  |  |
| HS_112 |  | 1.82 ^a^ |  |  |  |  |
| hsa-miR-181d |  | 1.60 ^a^ |  | 1.44 ^a^ |  |  |
| HS_147 |  | 1.63 ^b^ |  |  |  |  |
| HS_116 |  | 1.51 ^b^ |  |  |  |  |
| HS_7 |  | 1.55 ^a^ |  |  |  |  |
| hsa-miR-514 |  | 1.74 ^a^ |  |  |  |  |
|  |  | 1.76 ^b^ |  |  |  |  |
| hsa-miR-135b | -1.33 ^a^ |  | -1.63 ^b^ |  |  | -1.72 ^a^ |
|  |  | 1.83 ^b^ |  |  |  |  |
| hsa-miR-204 |  | 1.45 ^b^ |  | 1.40 ^a^ |  |  |
| hsa-miR-591 | -1.25 ^a^ |  |  | 1.40 ^a^ |  |  |
| hsa-miR-671:9.1 |  | 1.57 ^a^ |  |  |  |  |
| HS_120 |  | 1.57 ^b^ |  |  |  |  |
| hsa-miR-369-3p |  |  |  |  |  | -1.43 ^a^ |
| hsa-miR-1251 |  | 1.87 ^a^ |  |  |  |  |
| hsa-miR-603 |  | 1.49 ^a^ |  |  |  |  |
| hsa-miR-345:9.1 |  | 1.49 ^a^ |  |  |  |  |
| hsa-miR-208b |  | 1.63 ^a^ |  |  |  |  |
| HS_115 |  | 1.70 ^a^ |  |  |  |  |
| HS_111 |  | 1.78 ^a^ | 2.32 |  |  |  |
| HS_254 |  | 1.68 ^b^ |  |  |  |  |
| HS_76 |  | 1.82 ^b^ |  |  |  |  |
| HS_52 |  |  |  | 1.35 ^a^ |  |  |
| hsa-miR-517a,517b |  | 1.44 ^b^ |  |  |  |  |
| HS_45.1 |  | 1.70 ^a^ |  |  |  |  |
| hsa-miR-302a |  | 1.44 ^a^ | 1.50 ^a^ |  |  |  |
| HS_255 |  | 1.75 ^a^ |  |  |  |  |
| hsa-miR-328 | -1.42^b^ |  |  | 1.34 ^a^ |  |  |
| hsa-miR-876-3p | 2.14 ^b^ | 2.86 ^a^ | 2.14 ^b^ |  |  |  |
| hsa-miR-337:9.1 |  | 1.46 ^a^ |  |  |  |  |
| hsa-miR-590-5p |  | 1.41 ^a^ |  |  |  |  |
| HS_219 |  | 1.40 ^b^ |  | 1.33 ^a^ |  |  |
| HS_46 |  | 1.72 ^a^ |  |  |  |  |
| HS_240 |  | 1.60 ^a^ |  |  |  |  |
| HS_150 |  | 1.22 ^a^ |  | 1.32 ^a^ |  |  |
| HS_126 |  | 1.51 ^a^ |  |  |  |  |
| hsa-miR-518e*,hsa-miR-519a*,hsa-miR-519b-5p,hsa-miR-519c-5p,hsa-miR-522*,hsa-miR-523* |  | 1.67 ^a^ |  |  |  |  |
| HS_142.1 |  | 1.50 ^b^ |  |  |  |  |
| HS_80 |  | 1.66 ^a^ |  |  |  |  |
| HS_221 |  | 1.60 ^b^ |  |  |  |  |
| HS_35 |  | 1.53 ^a^ |  |  |  |  |
| HS_187 |  | 1.62 ^b^ |  |  |  |  |
| HS_179 |  | 1.54 ^a^ |  |  |  |  |
| hsa-miR-659 |  | 1.51 ^a^ |  |  |  |  |
| hsa-miR-412 |  | 1.48 ^a^ |  |  |  |  |
| hsa-miR-522 |  | 1.56 ^a^ |  |  |  |  |
| hsa-miR-302b |  | 1.42 ^a^ | 1.65 ^b^ |  |  |  |
| hsa-miR-571 |  | 1.42 ^a^ |  |  |  |  |
| HS_196.1 |  | 1.86 ^a^ |  |  |  |  |
| hsa-miR-95 | -1.35 ^a^ |  | -1.25 ^a^ |  |  |  |
| HS_25 |  | 1.41 ^a^ |  |  |  |  |
| HS_170 |  | 1.61 ^a^ |  |  |  |  |
| hsa-miR-375 | -1.51 ^a^ |  |  |  |  |  |
| HS_305_b |  | 1.35 ^b^ | 1.50 ^a^ |  |  |  |
| hsa-miR-604 |  | 1.45 ^a^ |  |  |  |  |
| HS_122.1 |  | 1.54 ^b^ |  |  |  |  |
| hsa-miR-581 |  | 1.34 ^a^ |  |  |  |  |
| hsa-miR-220a |  | 1.42 ^b^ |  |  |  |  |
| hsa-miR-520d-5p |  | 1.67 ^a^ |  |  |  |  |
| hsa-miR-92a | -1.30 ^a^ |  |  | 1.22 ^a^ |  |  |
| hsa-miR-509-3p |  | 1.47 ^a^ |  |  |  |  |
| hsa-miR-135a |  |  | -1.71 ^a^ |  |  | -1.56 ^a^ |
| hsa-miR-92b | -1.33 ^a^ |  |  | 1.21 ^a^ |  |  |
| HS_75.1 |  | 1.60 ^b^ |  |  |  |  |
|  |  |  |  |  |  |  |
| hsa-miR-613 |  | 1.52 ^a^ |  |  |  |  |
| HS_232 |  | 1.71 ^a^ |  |  |  |  |
| hsa-miR-647 |  | 1.60 ^b^ |  |  |  |  |
| HS_199 |  | 1.47 ^b^ |  |  |  |  |
| hsa-miR-33b |  | 1.51 ^a^ |  |  |  |  |
| hsa-miR-602 |  | 1.54 ^a^ |  |  |  |  |
| hsa-miR-564 |  | 1.31 ^a^ |  |  |  |  |
| HS_160 |  | 1.47 ^a^ |  |  |  |  |
| HS_19 |  |  |  | 1.18 ^a^ |  |  |
| hsa-miR-506 |  | 1.40 ^a^ |  |  |  |  |
| HS_119 |  | 1.69 ^b^ |  |  |  |  |
| HS_16 |  | 1.61 ^b^ |  |  |  |  |
| hsa-miR-129-5p |  |  |  | 1.17 ^a^ |  |  |
| hsa-miR-646 |  | 1.28 ^a^ |  |  |  |  |
| HS_86 |  | 1.58 ^a^ |  |  |  |  |
| HS_110 |  | 1.48 ^b^ |  |  |  |  |
| hsa-miR-186 |  |  |  | 1.16 ^a^ |  |  |
| HS_48.1 |  | 1.41 ^a^ |  |  |  |  |
| hsa-miR-645 |  | 1.30 ^a^ |  |  |  |  |
| hsa-miR-596 |  | 1.26 ^a^ |  |  |  |  |
| hsa-miR-517a |  |  |  | 1.14 ^a^ |  |  |
| hsa-miR-592 |  | 1.43 ^a^ |  |  |  |  |
| HS_141 |  | 1.33 ^a^ |  |  |  |  |
| hsa-miR-198 |  | 1.70 ^a^ | 1.99 ^b^ |  |  |  |
| hsa-miR-518d-3p |  | 1.29 ^a^ |  |  |  |  |
| hsa-miR-365 |  |  |  |  |  | -1.30 ^b^ |
| hsa-miR-200b |  |  |  |  |  | -1.27 ^a^ |
| HS_304_a |  | 1.51 ^a^ |  |  |  |  |
| hsa-miR-376a*:9.1 |  |  | -1.49 ^b^ |  |  |  |
| hsa-miR-191 |  | 1.44 ^b^ |  |  |  |  |
| hsa-miR-452*:9.1 | -1.23 ^a^ |  |  |  |  |  |
| HS_47 |  | 1.55 ^a^ |  |  |  |  |
| HS_208 |  | 1.53 ^a^ |  |  |  |  |
| hsa-miR-765 |  | 1.35 ^a^ |  |  |  |  |
| HS_262.1 |  | 1.49 ^b^ |  |  |  |  |
| hsa-miR-525-3p |  | 1.36 ^a^ |  |  |  |  |
| hsa-miR-144:9.1 | -2.32 | -2.10 |  |  | 2.71 | 2.45 |
| hsa-miR-329 | -1.19 ^a^ |  |  |  |  |  |
| hsa-miR-631 |  | 1.52 ^a^ |  |  |  |  |
| hsa-miR-122 | 2.03 | -1.86 |  |  |  |  |
| hsa-miR-128b:9.1 |  | 1.45 ^a^ |  |  |  |  |
| hsa-miR-508-3p |  | 1.55 ^a^ |  |  |  |  |
| hsa-miR-206 |  | 1.52 ^b^ |  |  |  |  |
| hsa-miR-548b-3p |  | 1.40 ^b^ |  |  |  |  |
| HS_279_a |  | 1.32 ^a^ |  |  |  |  |
| hsa-miR-517c |  | 1.26 ^a^ |  |  |  |  |
| HS_64 |  | 1.54 ^a^ |  |  |  |  |
| HS_268 |  |  |  |  | 1.18 ^a^ |  |
| hsa-miR-518c |  | 1.31 ^a^ |  |  |  |  |
| HS_269 | 1.45 ^a^ | 1.51 ^a^ |  |  |  |  |
| HS_40 |  | 1.44 ^a^ |  |  |  |  |
| hsa-miR-578 | 1.47 ^a^ | 1.53 ^b^ |  |  |  |  |
| hsa-miR-660 |  |  |  |  |  | -1.27 ^b^ |
| hsa-miR-134 |  |  | -1.38 ^b^ |  |  |  |
| hsa-miR-25 | -1.31 ^a^ | -1.27 ^a^ |  |  |  |  |
| hsa-miR-641 | 1.20 ^a^ | 1.23 ^a^ |  |  |  |  |
| hsa-miR-502-5p | 1.30 ^a^ | 1.33 ^a^ |  |  |  |  |
| hsa-miR-34b |  |  | -1.21 ^a^ |  |  |  |
| hsa-let-7g |  |  | 1.12 ^a^ |  |  |  |
| hsa-miR-30d | 1.39 ^a^ | 1.41 ^b^ |  |  |  |  |
| hsa-miR-502-3p |  |  | -1.22 ^a^ |  |  | -1.16 ^a^ |
| hsa-miR-155 | 1.22 ^a^ | 1.24 ^b^ | 1.23 ^a^ |  |  |  |
| hsa-miR-766 |  |  |  |  | -1.27 ^a^ | -1.28 ^a^ |
| hsa-miR-597 |  | 1.38 ^a^ |  |  |  |  |
| hsa-miR-30b | 1.22 ^a^ |  |  |  |  |  |
| HS_90 |  | 1.27 ^a^ |  |  |  |  |
| hsa-miR-450a |  |  | -1.37 ^a^ |  |  |  |
| hsa-miR-196a |  |  | -1.44 ^a^ |  |  |  |
| HS_113 |  |  | 1.23 ^a^ |  |  | 1.11 ^b^ |
| hsa-miR-93 | -1.29 ^a^ | -1.34 ^a^ |  |  |  |  |
| hsa-miR-299-3p |  | 1.47 ^b^ |  |  |  |  |
| hsa-miR-26b | 1.18 ^a^ |  | 1.18 ^b^ |  |  |  |
| HS_244 |  |  | -1.18 ^a^ |  |  | -1.14 ^a^ |
| hsa-miR-675 | -1.42 ^a^ |  | -1.33 ^a^ |  |  |  |
| hsa-miR-218 | 1.44 ^a^ |  |  |  |  |  |
| HS_252.1 | 1.41 ^a^ | 1.33 ^a^ |  |  |  |  |
| hsa-miR-30c | 1.06 ^a^ |  |  | -1.06 ^a^ |  |  |
| hsa-miR-455-5p |  | -1.26 ^a^ | -1.52 ^b^ |  | -1.29 ^a^ |  |
| hsa-miR-30e* |  | -1.18 ^a^ |  |  |  |  |
| hsa-miR-532-5p |  |  |  |  |  | -1.18 ^a^ |
| hsa-miR-801:9.1 |  |  | 1.66 ^a^ |  |  |  |
| hsa-miR-106b |  | -1.44 ^a^ |  |  |  |  |
| hsa-miR-609 | 1.43 ^a^ |  |  |  |  |  |
| hsa-miR-376a |  | 1.32 ^a^ |  |  |  |  |
| hsa-miR-221 |  |  |  | -1.11 ^b^ |  |  |
| hsa-miR-493 | 1.31 ^a^ |  |  |  |  |  |
| hsa-miR-607 | 1.42 ^a^ |  |  | -1.12 ^a^ |  |  |
| hsa-miR-503 | -1.19 ^b^ | -1.36 ^b^ | -1.38 ^b^ | -1.14 ^b^ | -1.15 ^a^ |  |
| has-miR-570 | 1.44 ^a^ | 1.24 ^a^ |  |  |  |  |
| has-miR-411 |  | -1.24 ^a^ | -1.21 ^a^ |  |  |  |
| has-miR-542-3p |  |  | -1.38 ^a^ |  |  |  |
| has-miR-142-5p |  |  |  | -1.19 ^a^ |  |  |
| hsa-miR-1296 |  |  |  |  | -1.38 ^a^ |  |
| hsa-miR-30e |  |  |  | -1.20 ^a^ |  |  |
| hsa-miR-449a |  | -1.86 ^a^ | -2.25 |  |  |  |
| hsa-miR-137 |  |  |  |  |  |  |
| hsa-miR-376c |  |  |  | -1.23 ^a^ |  |  |
| hsa-miR-326 | 1.57 ^a^ |  |  |  |  |  |
| hsa-miR-9 | 2.10 |  |  |  |  |  |
| hsa-miR-618 |  |  |  |  | -1.55 ^a^ |  |
| hsa-miR-15b |  |  |  | -1.29 ^a^ |  |  |
| hsa-miR-16 | 1.12 ^a^ |  |  | -1.30 ^a^ |  |  |
| hsa-miR-196b |  |  |  |  | -1.34 ^a^ |  |
| hsa-miR-146b-5p |  |  |  | -1.32 ^a^ |  |  |
| hsa-miR-100 |  |  |  | -1.34 ^a^ |  |  |
| hsa-miR-495 |  |  |  | -1.35 ^b^ |  |  |
| hsa-miR-20b |  | -1.43 ^a^ |  | -1.37 ^a^ |  |  |
| hsa-miR-485-3p |  |  |  | -1.37 ^a^ |  |  |
| hsa-miR-195 |  |  |  | -1.40 ^a^ |  |  |
| hsa-miR-15a |  |  |  | -1.50 ^a^ |  |  |
| hsa-miR-338-3p |  |  |  | -1.54 ^b^ |  |  |
| hsa-miR-486-5p |  | -2.19 |  | -1.57 |  | 2.53 |
| hsa-miR-146a |  |  |  | -1.58 ^a^ |  | 1.35 ^a^ |
| hsa-miR-99a |  |  |  | -1.64 ^a^ | -1.48 ^a^ |  |
| HS_176 |  |  |  | -2.15 |  | 2.76 |
| HS_97 |  | -2.13 |  | -2.39 |  | 3.03 |
| HS_203 |  | -2.13 |  | -2.60 |  | 3.41 |

^a^=*p*<0.05; ^b^=*p*<0.01
